# The role of pectoral nerve blocks in a day-case mastectomy service: A prospective cohort study

**DOI:** 10.1016/j.amsu.2019.10.019

**Published:** 2019-10-25

**Authors:** Ashleigh Bell, Oroog Ali, Amy Robinson, Amitabh Aggarwal, Michael Blundell, Alice Townend, Sebastian Aspinall

**Affiliations:** Northumbria NHS Foundation Trust, North Tyneside General Hospital, Rake Lane, North Shields, Tyne and Wear, NE29 8NH, UK

**Keywords:** Ambulatory surgery, Breast cancer, Nerve block, Mastectomy, Simple, Pectoralis muscles

## Abstract

**Background:**

It is now recognised that the majority of breast surgery can be safely undertaken as day case procedures. We aimed to evaluate the effect of pectoral nerve (Pecs2) blocks on recovery parameters and day case rates in patients undergoing mastectomy for breast cancer.

**Methods:**

A prospective cohort study was performed in a single NHS Foundation trust between 1st April 2014 and 31st December 2016. Visual analogue scale (VAS) pain scores (0–10) at 4 and 8 h, episodes of post-operative nausea ± vomiting (PONV), opioid use and day case outcome were compared between Pecs2 and no Pecs2 groups.

**Results:**

22 patients underwent general anaesthesia (GA) + Pecs2 block and 30 GA ± local anaesthetic infiltration.

Mean pain scores were significantly lower in the Pecs2 (2.5) vs no Pecs2 (4.6) group at 4 h (p = 0.0132) and 8 h, Pecs2 (1.9) vs no Pecs2 (3.6) (p = 0.0038).

Episodes of PONV requiring additional anti-emetic were lower and statistically significant in the Pecs2 group (2/22, 9%) than the no Pecs2 group (14/30, 46%), (p = 0.005).

Additional opioid use was significantly lower in the Pecs2 group (4/22, 18%) than in the no Pecs2 group (14/30, 46%) (p = 0.0423).

18 patients in the Pecs2 group were discharged the same day in contrast to just 3 patients in the no Pecs2 group. This was highly statistically significant (p = 0.0001).

**Conclusions:**

Pecs2 blocks can significantly reduce post-operative pain, nausea and vomiting in patients undergoing mastectomy. Their use can enable units to achieve high day-case mastectomy rates.

## Highlights

• Pecs2 blocks provide good post-operative pain control in mastectomy.
• Pecs2 blocks reduce levels of nausea compared with GA ± LA.
• Pecs2 block can help facilitate a high day case rate for mastectomy.

## 1 Introduction

Breast cancer management has evolved greatly since the mid 1980's when patients would typically have stayed in hospital for up to ten days following surgery. Incidence rates for breast cancer are projected to rise by 2% in the UK in the next two decades with a need to limit inpatient costs in the face of an increasing workload [1]. Whilst the majority of breast conserving surgery is already performed on a day case basis, the superficial nature of mastectomy also makes these patients ideal candidates for day case surgery.

A major factor in successful same day discharge is the technical ability of the attending anaesthetist. In some centres, general anaesthesia alone, or in combination with local anaesthetic infiltration of the skin continues to be the mainstay of analgesia.

New ultrasound-guided inter-fascial regional anaesthesia techniques for the thorax are available with evidence for their opioid sparing effects, reduction in post-operative nausea and vomiting (PONV) and earlier mobilisation of patients [2].

The Pectoral nerve (Pecs1) block involves a hydro-dissection of the fascial plane between the pectoral muscles with local anaesthetic to block the lateral and medial pectoral nerves. The block is performed with the patient supine, either with the arm parallel to the chest or abducted 90°. The suggested volume is 0.2 ml/kg of a long acting local anaesthetic. The Pecs2 block is an extension of this technique and involves a second injection lateral to the Pecs1 injection point in the plane between the pectoralis minor and serratus anterior muscles. This provides blockade of the lateral cutaneous branches of the intercostal nerves that supply the chest wall and is therefore an effective analgesic technique in mastectomy [3].

We present our experience of using Pecs2 blocks in symptomatic patients undergoing mastectomy as a method for facilitating same day discharge.

## 2 Methods

### 2.1 Patient population

This prospective study describes a cohort of patients who underwent a mastectomy for treatment of breast cancer between 01/04/14 and 31/12/16. All patients were treated within the same NHS Foundation Trust at 3 district general hospitals. Inclusion criteria were all patients, over the age of 18, undergoing unilateral mastectomy (+/- axillary procedure) under general anaesthesia. Patients undergoing breast reconstruction as part of the same procedure were excluded.

### 2.2 Methods

Local approval for the study was obtained at a trust level. After screening for eligibility, information on the study was provided to patients by the operating surgeon at the pre-operative clinical visit with written informed consent taken and recorded in the patient's notes.

Patients were not randomly assigned to the Pecs2 or no Pecs2 groups. The decision to perform a Pecs2 block or not was made by the anaesthetic team on the day of surgery.

Post-operative data was recorded by the surgical team in charge of care or nursing staff on the evening or first post-operative day either by phone (for day-case patients) or in person (for in-patients).

Primary outcomes were visual analogue scale (VAS) pain scores (0–10) at 4 and 8 h, incidence of episodes of post-operative nausea ± vomiting (PONV) requiring additional anti-emetic, incidence of episode requiring additional administration of an opioid and day case outcome.

### 2.3 Technique for Pecs2 block

Verbal consent for the Pecs2 block was obtained during the pre-operative anaesthetic review on the day of surgery. The block was performed under ultrasound guidance using a 22G 80/100 mm Pajunk needle in the supine position following commencement of GA. 10 ml of Levobupivicaine was infiltrated for the Pecs1 block and 20 ml for the Pecs2 block. The deeper injection of the Pecs2 block was a sub serratus injection achieved with the needle on the exterior surface of rib 4. An additional para midline subcutaneous infiltration was used to cover the anterior perforating branch of the intercostal nerve plus contra-lateral nerve supply over the medial aspect of the mastectomy incision.

### 2.4 Surgical technique

All sentinel lymph node biopsies (SLNB) were performed using a dual technique. All mastectomies were performed using a scalpel for incisions and mono-polar diathermy for raising the upper and lower flaps.

### 2.5 Statistics

Data was collected and handled using a standardised spreadsheet (Microsoft Excel 2010) and analyses undertaken using IBM SPSS Statistics V22 software. Results are presented as a mean with a 95%.

Confidence interval for continuous variables and in numbers and percentages for categorical variables.

Differences between groups were compared using Fisher's exact test for categorical variables and students *t*-test for continuous variables. Statistical significance was defined as P < 0.05.

### 2.6 Ethics

Local approval was obtained at the authors institute.

### 2.7 Reporting

Our findings are reported in line with STROCSS criteria [4].

## 3 Results

Between 01/04/14 and 31/12/2016, 52 consecutive patients were recruited in to the study.

There was no significant difference in age between the two groups with a mean age of 64 years in the no Pecs2 group and 61 years in the Pecs2 group (p = 0.925). The majority of patients were ASA 2 with no significant difference in ASA grade between the two groups (p = 0.953) (Table 1). All patients were female.

**Table 1:** Patient demographics/clinical stage.

	No Pecs2 (n = 30)	Pecs2 (n = 22)	p value
Mean age (mean, 95% CI)	64 (59.2, 70.9)	61 (56.1, 70.2)	0.925
ASA 1	5	4	0.953
2	20	15	
3	5	3	
4	0	0	
Clinical T stage T1/T2/T3/T4	5/18/7/0	4/17/1/0	0.175
Clinical stage of axilla N0/N1	19/11	6/16	0.010

There were similar numbers of clinical T stage between the two groups (p = 0.175). There was a higher proportion of clinically positive axilla's in the Pecs2 group. (p = 0.010).

22 patients received a Pecs2 block. Of the 30 patients in the no Pecs2 group, 24 patients received additional long-acting local anaesthetic (Levobupivicaine) infiltrated under the skin. 3 patients received a pain buster consisting of a continuous infusion of Levobupivicaine via an elastomeric pump and wound infiltration catheter. 3 patients had GA only.

1 patient in each group required simple mastectomy for completion for involved margins. These were performed using an elliptical or IMF based incision with no axillary procedure. There were similar numbers of patients undergoing mastectomy with SLNB but there was a higher proportion of patients who underwent modified radical mastectomy in the noPecs2 group 16/30 (53%) than in the Pecs2 group (31%) (Table 2).

**Table 2:** Surgical procedure.

	No Pecs2 (n = 30)	Pecs2 (n = 22)	p value
Simple mastectomy (SM) - no additional axillary procedure	1 (23%)	1 (6%)	0.304
Mastectomy + sentinel lymph node biopsy (SLNB)	13 (43%)	14 (63%)	
Modified radical mastectomy (incl. level I + II clearance)	16 (53%)	7 (31%)	

The average duration of surgery was higher in the noPecs2 group at 98 min compared with 76 min in the Pecs2 group.

There was a slightly higher proportion of patients with drains left in situ in the no Pecs2 group (29/30, 96%) vs Pecs2 group (19/22, 86%) though this was not statistically significant (p = 0.298).

Mean pain scores were significantly lower in the Pecs2 (2.5) vs. no Pecs2 (4.6) group at 4 h (p = 0.0132) and 8 h Pecs2 (1.9) vs. no Pecs2 (3.6) (p = 0.0038) (Table 3).

**Table 3:** Mean pain scores at 4 h and 8 h.

	No Pecs2	Pecs2	p value
Pain score 4 h (mean + 95% CI)	4.6 (3.61–5.65)	2.5 (1.31–3.79)	p = 0.0132*
Pain score 8 h (mean + 95% CI)	3.6 (2.84–4.29)	1.9 (0.99–2.60)	p = 0.0038*

**Fig. 1:**
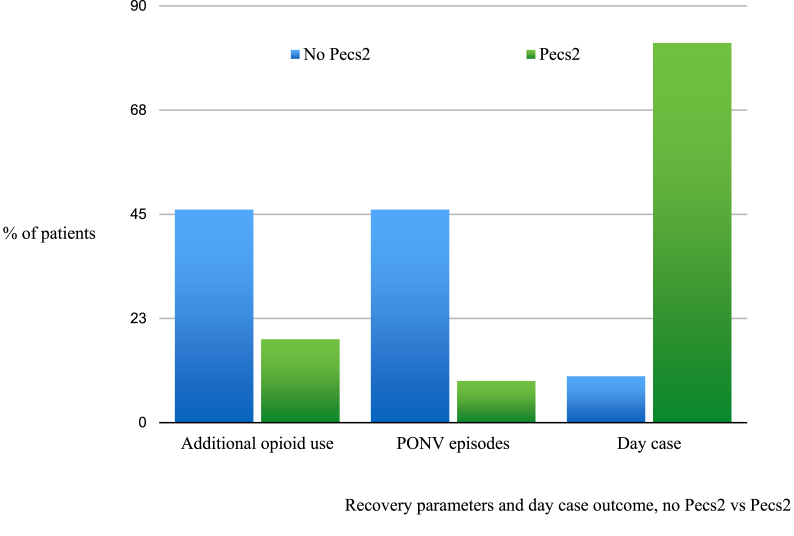
Recovery parameters and day case outcome, no Pecs2 vs Pecs2.

The number of patients requiring additional post-operative opioid use was lower in the Pecs2 group (4/22, 18%) than in the no Pecs2 group (14/30, 46%) (Fig. 1). This was statistically significant (p = 0.0423). 1 patient in each group required intravenous patient-controlled analgesia on the first post-operative night.

Episodes of PONV requiring administration of an anti-emetic agent were lower and statistically significant in the Pecs2 group (2/22, 9%) than in the no Pecs2 group (14/30, 46%), p = 0.0055 (Fig. 1).

No complications related to the Pecs2 block technique were observed in any patient in our study.

Day case rates were higher in the Pecs2 group (18/22, 82%) than in the no Pecs2 group (3/30, 10%). This was statistically significant (p = 0.0001) (Fig. 1). There were no unexpected re-admissions to hospital within 30 days of surgery.

## 4 Discussion

We have demonstrated that in patients undergoing mastectomy for breast cancer, Pecs2 blocks offer superior levels of post-operative pain and nausea control when compared with GA alone or in combination with LA skin infiltration.

These findings support those found in a number of other studies including Versyck et al. who demonstrated significantly less post-operative opioid consumption with additional Pecs2 blocks following mastectomy or wide local excision [5]. Bashandy also reported statistically significant lower pain scores, intra-operative fentanyl use and opioid consumption in patients undergoing SM with Pecs2 block when compared with GA alone. In this study however, there was an inadequate description of allocation concealment [6].

There remains conflicting evidence as to which of the regional block techniques is superior. Kulhari reported a significantly prolonged duration of analgesia in radical mastectomy patients receiving a Pecs2 block (4.9 h) compared with TPVB (3.9 h), with no difference in incidence of adverse side effects between the two groups [7]. Syal et al. however found mean duration of analgesia was significantly prolonged in the group receiving TPVB compared with Pecs2 [8]. A recent 2017 review concluded that TPVB's produce a higher overall reduction in length of stay but suggests an even greater benefit could be achieved by using TPVB and a Pecs2 in combination [9].

We are aware of the theoretical risk that Pecs blocks could reduce sensitivity of blue dye for sentinel lymph node biopsy with the potential for trauma to lymphatics in the sub pectoral or inter pectoral plane. However, in most instances, the sentinel node receives lymph from more than one lymphatic vessel thereby minimising this risk, especially when performed as a dual technique.

Our study provides level 2b evidence that Pecs2 blocks are effective. The study was performed in a non-screening centre and we acknowledge that the relatively small sample size may not be truly representative of all patients undergoing mastectomy for breast cancer. All Pecs2 blocks were performed in lists led by a single anaesthetic consultant. No randomisation method was used to allocate patients to either receive the block or not. We cannot discount the possibility that this introduced a systematic bias, however no attempt was made to steer patients more suitable to day-case surgery towards an operating list undertaken by the anaesthetist proficient in its use. It is also possible that the surgical/anaesthetic team which used the Pecs2 blocks were more motivated to discharge patients on the same day than the teams that didn't with no standard discharge criteria used. 27 of the 30 patients in the noPecs2 groups received additional LA either in the form of simple skin infiltration or via a continuous infusion. Failure rate of the technique could not be assessed as all Pecs2 blocks were performed following commencement of GA. It is difficult to ascertain what effect (if any) these factors had on pain scores between the two groups and could potentially have introduced bias into interpretation of the results.

We also acknowledge that the groups were slightly different in clinical stage of the axilla and requirement for modified radical mastectomy. In patients with no complete axillary dissection, pain is likely to be less severe regardless of whether there is use of a nerve block or not. Future studies should aim to stratify groups or assess this technique as a larger randomised controlled trial.

Despite prominence in anaesthetic literature, we have highlighted a significant gap in training. The desire to move away from the more traditional approaches of thoracic epidural and thoracic para-vertebral blocks (TPVB) is in part due to their required specialist skills and potential for serious adverse complications. If training needs were addressed, wider use of this simpler technique could become routine practice.

Another barrier to wider adoption is awareness and proactivity of the breast surgeon in ensuring its use. It is arguably therefore just as important to include in surgical training pathways as it is in anaesthesia with similar efficacy results seen with intra-operative as opposed to peri-operative use [10].

Placement of drains also plays a role in successful same day discharge. Many patients express a desire to stay in hospital until drains are removed and with poor evidence for their prevention of post-operative seroma [11], drain use should be audited on a national level. In our study, the decision to leave a drain was left to the discretion of the operating surgeon with no significant difference in the rate of drain placement between the two groups.

Social factors too constitute a considerable variable. Our trust serves a population of in excess of 500,000 over a very wide geographical area and includes some of the most rural parts of England. The effect of distance from hospital to home and home circumstances were not specifically accounted for. It is clear that thorough discussion and effective goal setting in the pre-operative stage is paramount in allaying anxiety and ensuring patients have realistic expectations of same day discharge.

In an otherwise homogenous population of patients, we have demonstrated that the additional use of regional anaesthesia, in this case a Pecs2 block can significantly reduce levels of pain and PONV following mastectomy. This has helped us facilitate a day case rate of 82%, well above the target of 50% set by the British Association of Day Case Surgery [12]. Wider adoption and training of this approach, regardless of technique, is needed to provide a fully day case breast service.

## Ethical approval

Local approval was obtained at the authors institute.

## Sources of funding

No external funding or sponsorship was received by any author for the conduct of the study.

## Author contribution

A Bell - Formal analysis, project administration, writing - original draft.

O Ali - Data curation, formal analysis.

A Robinson - Conceptualisation, data curation.

A Aggarwal - Data curation, formal analysis.

M Blundell - Conceptualisation, supervision, writing - review and editing.

A Townend - Supervision.

S Aspinall - Conceptualisation, supervision, writing - review and editing.

## Consent

Local approval was obtained at the authors institute.

Fully informed consent was taken at the pre-operative surgical visit.

## Registration of research studies

Name of the registry: Research Registry.

Unique Identifying number or registration ID: Researchregistry4825.

Hyperlink to the registration (must be publicly accessible): https://www.researchregistry.com/browse-the-registry#home/registrationdetails/5cbeec8eb272eb2e718a6a5d/

## Guarantor

Ashleigh Bell.

Sebastian Aspinall.

## Provenance and peer review

Not commissioned, externally peer reviewed.

## Declaration of competing interest

None.
